# Turkestan Cockroaches Avoid Entering a Static Electric Field upon Perceiving an Attractive Force Applied to Antennae Inserted into the Field

**DOI:** 10.3390/insects12070621

**Published:** 2021-07-08

**Authors:** Yoshinori Matsuda, Teruo Nonomura, Hideyoshi Toyoda

**Affiliations:** 1 Laboratory of Phytoprotection Science and Technology, Faculty of Agriculture, Kindai University, Nara 631-8505, Japan; nonomura@nara.kindai.ac.jp; 2 Research Association of Electric Field Screen Supporters, Nara 631-8505, Japan; toyoda@nara.kindai.ac.jp

**Keywords:** avoidance behaviour, electron deprivation, repulsive force, transient electric current, negative voltage generator, pole distance

## Abstract

**Simple Summary:**

Electrostatic devices that generate a static electric field (S-EF) are used as barriers to repel insect pests because insects habitually avoid entering a S-EF. Understanding the avoidance mechanism would provide an experimental basis for developing electrostatic-based pest control methods. An apparatus generating a S-EF was constructed by placing a polyvinyl chloride-insulated iron plate (N-PIP) that was negatively charged using a voltage generator parallel to a grounded metal net (G-MN) that was positively polarised via electrostatic induction due to the negative charge of the N-PIP. The S-EF formed in the space between the N-PIP and G-MN, where the negative charge of the N-PIP generated a repulsive force to push electrons in the field toward the ground via the G-MN. A test insect (an adult Turkestan cockroach, *Shelfordella lateralis* Walker) released in the space surrounded by the apparatus inserted its antenna into the S-EF and reflexively moved backward. Free electrons were pushed out of the antenna tip toward the ground, and the antenna became positive. The positively polarised antenna was attracted to the N-PIP, and in response to this force, the insect pulled its antennae back reflexively and moved backward. This insect action was recognised as avoidance behaviour.

**Abstract:**

This study analysed the mechanism of avoidance behaviour by adult Turkestan cockroaches (*Shelfordella lateralis* Walker) in response to a static electric field (S-EF) formed in the space between a negatively charged polyvinyl chloride-insulated iron plate (N-PIP) and a grounded metal net (G-MN). The negative surface charge supplied to the iron plate by a voltage generator caused the G-MN to polarise positively via electrostatic induction. In the S-EF, the negative charge of the N-PIP created a repulsive force that pushed free electrons in the field toward the ground via the G-MN. When insects released in the space surrounded by the S-EF inserted their antennae into the S-EF, they pulled them back reflexively and moved backward. The analysis indicated that an electric current flowed transiently toward the ground when an insect inserted its antennae into the S-EF. The insect became positively charged via this discharge and was attracted to the opposite pole (N-PIP). In response to this attractive force, the insect pulled its antennae back quickly. The positive electrification caused by the removal of free electrons from the antenna tip triggered the avoidance behaviour.

## 1. Introduction

Insects often avoid entering a static electric field (S-EFs) [1] due to their ability to perceive S-EFs. Nonomura et al. [2] reported that whiteflies inserted their antennae into the S-EF formed between the negatively charged insulated conductor and grounded metal net and were deterred from entering the S-EF. Newland et al. [3] reported that cockroaches can detect an electrostatic filed (EF) with their antennae. The cockroaches placed in the EF deflected their antennae against the attractive forces and then moved them toward the electrode. The antennae appear to be influenced by the electrostatic force, which leads to an uneven distribution of free electrons in the organ as a result of electrostatic induction [3].

An electric field is defined as the space surrounding an electric charge within which it is capable of exerting a perceptible force on another electric charge [4]. Electrifying a conductor is a primary step to form an electric field. A grounded negative voltage generator is used for this purpose, by which free electrons (negative charges) are drawn from the ground and supplied to the conductor. These negative charges accumulate on the conductor surface [5]. Using an insulated conductor, the insulated coating is dielectrically polarised by the negative charges on the conductor, negatively electrifying the outer surface of the coating [6]. The amount of negative charge for electrification is proportional to the voltage applied by the negative voltage generator, which corresponds to the potential difference against the earth ground [6].

A charged, insulated conductor used as a single pole forms the EF in its surrounding space [7], where the negative charge on the conductor is not discharged in the field. On the other hand, a pair of negatively charged, insulated conductor and grounded conductor poles creates an electric field between them, where the charged conductor causes an electric discharge toward the grounded conductor if the voltage applied exceeds a limit. This type of electric field is specified by the presence or absence of a discharge; a non-discharging electric field is referred to as the S-EF and a discharge-causing electric field as a dynamic electric field [7].

The S-EF generates sufficient attractive or repulsive force, by which spores and insect pests entering the electric field are captured [8]. This electric field is characterised by the negative charge of the insulated conductor, which generates a strong repulsive force against other negative charges (free electrons) in the S-EF, pushing them toward the ground. Via this mechanism, any conductor in the S-EF is deprived of its free electrons and becomes positively charged. This phenomenon is called discharge-mediated positive electrification of the conductor [8]. Insects that enter S-EFs are similarly electrified positively and attracted to the negatively charged insulated conductor [9,10]. This force is so strong that the captured insects are not able to escape. This mechanism was able to capture all 15 insect species tested [10].

High-voltage electric fields can be used as control measures for biotic and abiotic environmental nuisances. In fact, the strong attractive forces generated in the EF or S-EF were harnessed to develop an electric field screen to prevent entry of the airborne spores of pathogens [11,12,13], flying insects [14,15], pollen grains that cause pollenosis [16] and fine smoke particles [17]. In addition to insect capturing function, the electric field screen repelled insects that inherently avoid entering the S-EF [1,2,15]. Insects exhibiting this avoidance behaviour have been reported from 82 species in 17 orders, 42 families and 45 genera [1]. This study sought to clarify the mechanism of the insect avoidance behaviour against the S-EF.

Our interest was to clarify the relationship between the insect avoidance behaviour against the S-EF and the release of negative electricity from the insect antennae that were inserted into the S-EF. In the previous works, we attempted to detect an electric current generation from the antennae of the test insects, such as cigarette beetles (*Lasioderma serricorne*) [15], whiteflies (*Bemisia tabaci*) [2] and rice weevils (*Sitophilus oryzae*) [18]. However, these approaches were unsuccessful due to low amounts of electric current below the detection limit. Kakutani et al. [10] reported that larger insects that were transferred into the S-EF released larger amounts of electric current. This result strongly suggested that if we use much larger insects, we can detect sufficient amounts of transient electric current from the antennae when inserted into the S-EF. From this view point, we selected adult cockroaches for investigation.

This study examined whether the Turkestan cockroach (*Shelfordella lateralis* Walker) (Blattodea: Blattidae) can avoid the S-EF and clarified the importance of the attractive force applied to their antennae, which were inserted into the S-EF; the insects instantly withdrew their antennae in response to the attractive force. Importantly, this work reveals that this attractive force is due to the instantaneous, transient removal of free electrons (positive electrification) from the antennae via a repulsive force in the S-EF.

In this study, we fabricated an electrostatic apparatus to create the S-EF, S-EF producer (S-EFP), using a negatively charged polyvinyl chloride (PVC)-insulated iron plate (N-PIP) and a grounded stainless steel net (conductor) (G-MN). Then, we examined the avoidance behaviour of adult male and female Turkestan cockroaches in response to the S-EF when they generated a detectable electric current from their antennae inserted into the electric field.

## 2. Materials and Methods

### 2.1. Insects

Adult male and female Turkestan cockroaches were purchased from Prosper International (Tokyo, Japan) and maintained in a diet (rabbit food pellets)-containing container (25 cubic centimetres) in a growth chamber (25.0 ± 0.5 °C, 12-h photoperiod at 4000 lux). Male and female cockroaches (50 adults per container) were separately maintained in different containers until use. To slow their movement, the cockroaches were kept at −10 °C for 10 min and transferred using bamboo tweezers with rubber-capped tips. Within 3–5 min after transfer, the insects recovered to their initial state. Figure S1 shows the size of these insects.

### 2.2. Constructing the S-EFP and Detecting a Transient Electric Current from an Insect

**Figure 1:**
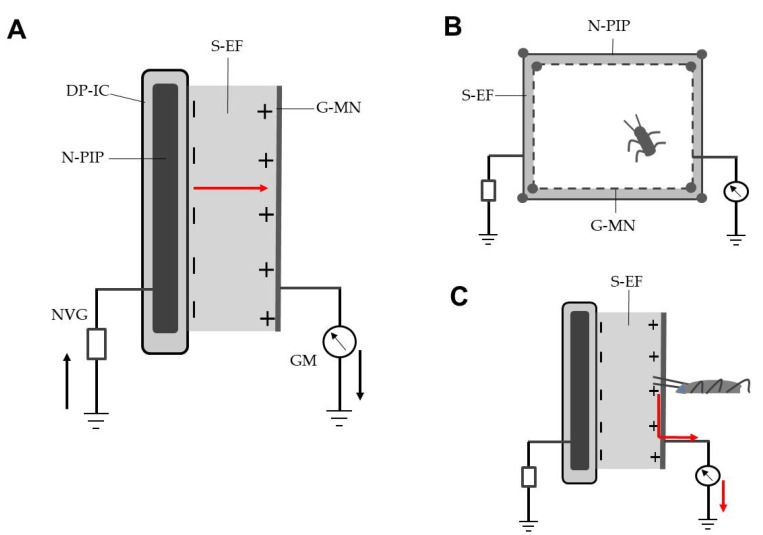
(**A**) Schematic representation of the static electric field producer (S-EFP) (cross-sectional view). The solid arrow denotes electron movement from electrode to ground via mechanical discharge (red arrow) between opposite poles. (**B**) A square surrounded by four S-EFPs was used to examine the insect avoidance behaviour (bird’s-eye view). (**C**) Flow of a transient electric current (red arrow) from an insect that inserted its antennae into the static electric field. Abbreviations: DP-IC, dielectrically polarised insulator coating with negative charge on outer surface; N-PIP, negatively charged polyvinyl chloride-insulated iron plate; NVG, negative voltage generator; S-EF, static electric field; G-MN, grounded metal (stainless steel) net; GM, galvanometer.

Figure 1A shows the S-EFP, which consisted of N-PIPs (PVC membrane thickness, 1 mm; membrane resistivity, 10^9^ Ωcm; plate size, 20 × 200 mm^2^) (Sonoda Seisakusho, Osaka, Japan) linked to a direct-current negative voltage generator (highest voltage available, −10 kV) (Max Electronics, Tokyo, Japan) and a same-sized stainless steel net (mesh size, 1.5 cm) linked to ground lines that were arranged vertically in parallel at 9-mm intervals. The N-PIP was negatively charged with different voltages. The negative surface charge of the N-PIP positively polarised the grounded metal net (G-MN) via electrostatic induction. A static electric field formed in the space between the negatively charged N-PIP and G-MN. A galvanometer (Sanwa, Tokyo, Japan) was integrated in the ground line of the G-MN to measure the current to the ground via the grounded plate. The electric current profiles were recorded with a current detector (detection limit, 0.01 µA) integrated into the galvanometer.

In the present experiment, the voltage applied to the N-PIP of the S-EFP was raised gradually to determine the lowest voltage that caused a discharge from the charged plate (i.e., current from the charged insulated conductor to the grounded metal net via silent discharge) [19] in the absence of a test insect (Figure 1A). In subsequent experiments, insect avoidance assays were conducted using voltages of −1 to −6 kV that caused no mechanical discharge.

### 2.3. Insect Avoidance Assay

In the first experiment, the insulated plate of the S-EFP was negatively charged with different voltages, and test insects were released inside an area surrounded by S-EFPs to examine whether they avoid entering the S-EF (Figure 1B). The insect avoidance behaviour was recorded on video, while the transient electric current produced upon inserting antennae into the S-EF was recorded by the current detector in the galvanometer (Figure 1C). In the second experiment, the ground line of the galvanometer was removed to impede the current flow to the ground, and then an insect was similarly released inside the square to examine whether it showed avoidance. In both experiments, 20 male and 20 female adults were used.

### 2.4. Statistical Analysis

All experiments were repeated five times, and data are presented as the means and standard deviations. Tukey’s test and linear regression analysis, using EZR software ver. 1.54 (Jichi Medical University, Saitama, Japan), were used to identify significant differences among the conditions and correlations among the factors, which are shown in the figure and table legends.

## 3. Results and Discussion

The first aim of this study was to construct an electrostatic apparatus suitable for the purpose of the present work. A negatively charged insulated conductor wire dielectrically polarises the insulating coating [6], negatively charging the insulator surface; the charged insulator surface produces the EF in the surrounding space [8]. If a G-MN is placed inside the EF produced by the insulated conducting wire, the G-MN facing the charged insulated conductor becomes positively charged as a result of electrostatic induction; eventually, the negative insulator surface pushes the free electrons in the metal net to the ground [8], by which the metal net leaves a positive charge. The opposite charges of the negatively charged insulated conductor wire and positively polarised G-MN generate a dipole, forming an electric field in the space between them. If we select a voltage range that causes no discharge from the charged insulated conductor wire, the poles create a S-EF between them. In this study, we found that the N-PIP was most effective to form a uniform distance between the charged plate and G-MN (Figure 1A).

This work sought to clarify how a cockroach senses the S-EF through its antennae using video-based analysis of the insect behaviour to assess the generation of an attractive force on the antenna and its reflex movement. When an insect inserted its antenna tips into the S-EF slightly, it quickly pulled them back (Video S1A). This action was repeated by adult males and females released in the space surrounded by the S-EFP (see Figure 1B). We detected the transient electric current that occurred when an insect inserted its antennae into the field using cockroaches, which are considerably larger than previous test insects, such as the cigarette beetle (*Lasioderma serricorne*) [15], whitefly (*Bemisia tabaci*) [2] and rice weevil (*Sitophilus oryzae*) [18], which have respective body lengths of 2.95 ± 0.28, 0.79 ± 0.21 and 2.56 ± 0.27 mm. The transient electric currents from these insects were too small to detect.

**Figure 2:**
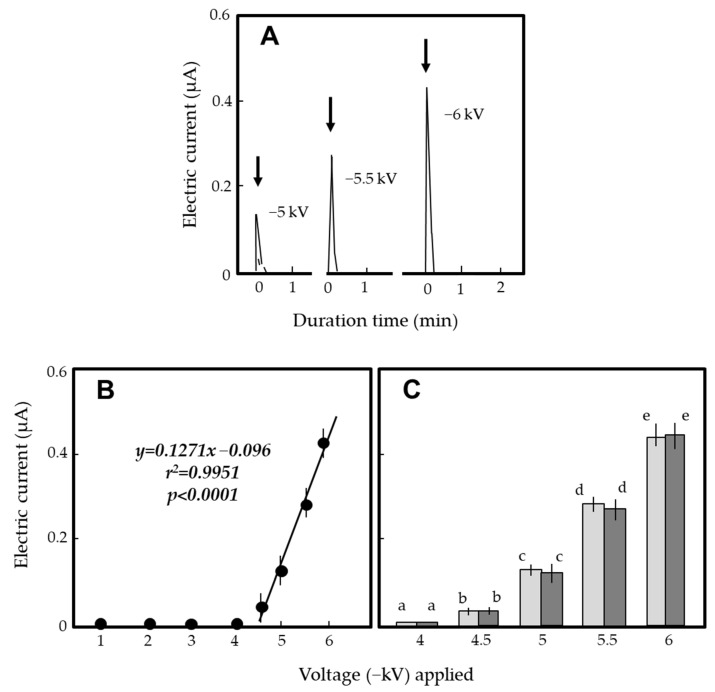
(**A**) Profiles of transient electric currents from discharges of adult female Turkestan cockroaches that inserted their antennae into the static electric field of the S-EFP negatively charged with different voltages. (**B**) Relationship between the applied voltage and magnitude of the transient electric current. (**C**) Difference in the magnitude of the electric current between adult male (left) and female (right column) cockroaches. Twenty insects were used per voltage. The means and standard deviations were calculated from five repetitions of the experiments. The letters (a–e) attached to the columns indicate significant differences (*p* < 0.05) according to Tukey’s method.

**Figure 3:**
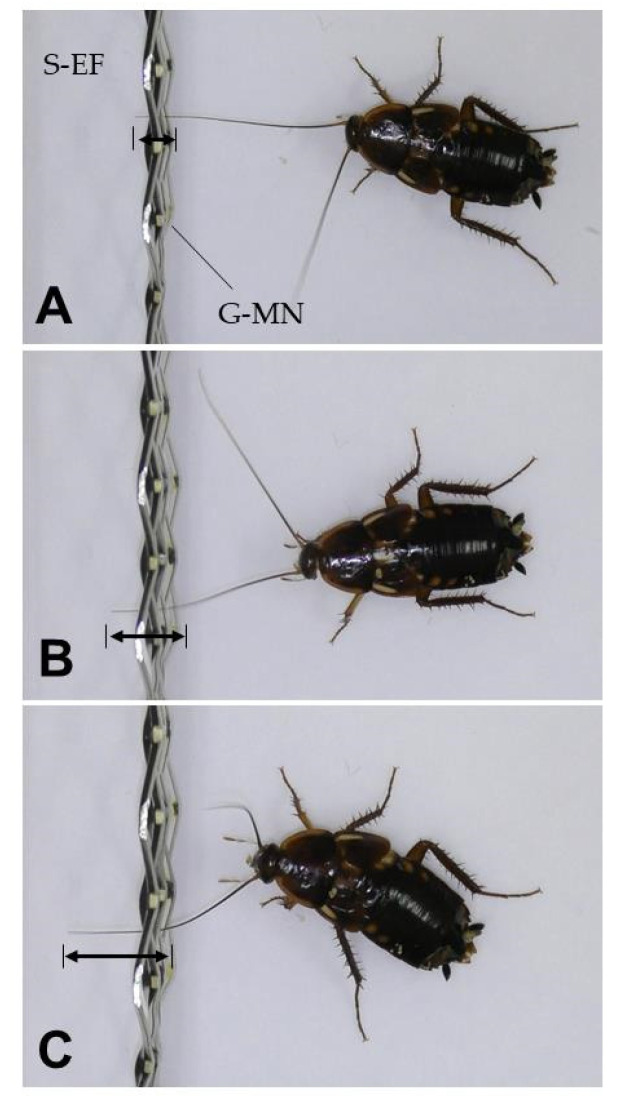
Insertion of the antennae by adult female Turkestan cockroaches into the static electric field (S-EF) of the S-EFP charged with −6.0 (**A**), −4.9 (**B**) and −4.5 kV (**C**). The arrow shows the distance the antenna inserted from the grounded metal net (G-MN).

Figure 2A shows the magnitude of the transient electric current produced upon inserting an antenna into the S-EFP static electric field negatively charged with different voltages. In the range of −5 to −6 kV, a transient electric current was generated as soon the antennae were inserted 1–2 mm inside the static electric field (Figure 3A). The regression analysis clearly indicated that the current magnitude increased linearly with the voltage applied with high correlation (Figure 2B). In both male and female adults, there was a significant difference in the transient electric current generated among the voltages applied (Figure 2C). On the other hand, there was no significant difference in the transient current generated by adult male versus female Turkestan cockroaches (Figure 2C).

At lower voltages (−4.0 to −4.9 kV), the antennae were subjected to the attractive force of the N-PIP when they were inserted much deeper (3–8 mm) (Figure 3B,C). The insertion distance increased as the voltage applied decreased. In this range, despite the insect’s avoidance behaviour, no electric current was detected, probably because the current magnitude was below the detection limit. At <−4.0 kV, the insect showed no avoidance reaction.

When the ground line of the galvanometer was removed, no avoidance behaviour was detected (Video S1B), regardless of the voltage applied, indicating that the current flow from the insect to the ground was essential for the insect to be positively polarised (i.e., subjected to the attractive force of the N-PIP). We concluded that greater voltages pushed more negative electrons out of the insect antenna, which caused the insects to have a greater positive charge and to be attracted more strongly by the oppositely charged insulated plate.

**Figure 4:**
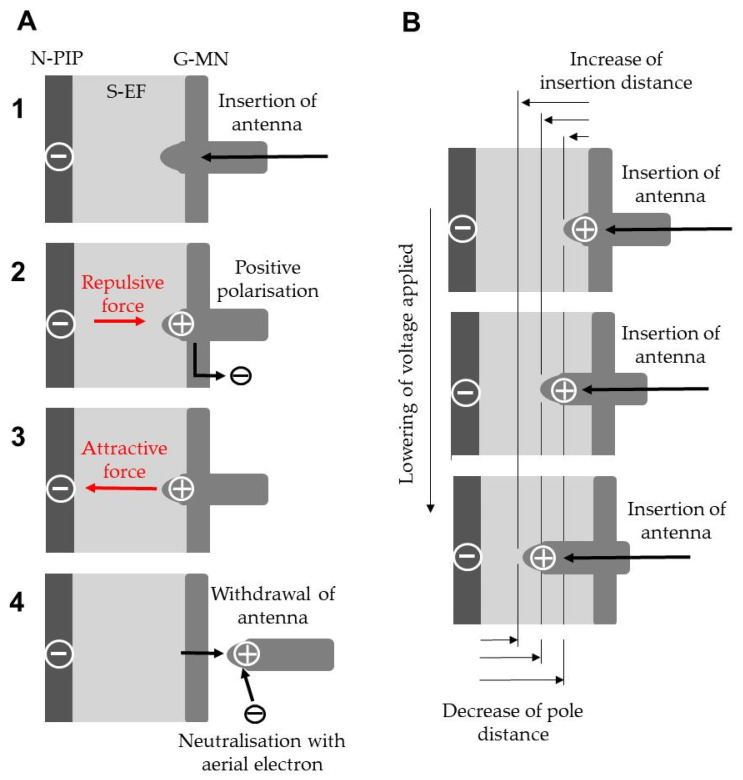
(**A**) Schematic representation of the electrostatic and biological events that cause the insect to avoid entering the static electric field (S-EF). (**B**) Relationship between lowering the applied voltage and increasing the insertion distance of the antennae into the static electric field or the decrease in the pole distance (interval between the negatively charged insulated plate (N-PIP) and the tip of the antenna that touched the grounded metal net (G-MN)).

Figure 4 schematises the series of events that caused the insect to avoid the static electric field. The initial event was the action of the antennae searching the space in front of the insect [2] by inserting the antenna into the space (Figure 4A1). The discharge from the insect indicated positive electrification of its body. The 1.5-cm-mesh G-MN was narrow, and one or both antennae inevitably touched the string of the G-MN when they were inserted; free electrons in the touched region were pushed out toward the ground via the G-MN (Figure 4A2). The tip of the antenna was positively charged and then attracted to the opposite charge of the N-PIP (Figure 4A3). As shown in Video S1A, the insect pulled its antennae back reflexively and moved backward (Figure 4A4). It was likely that the positively polarised antennae attracted free electron in the air [20] to restore the original state via neutralisation when they were pulled back out of the S-EF (Figure 4A4).

The change in the voltage applied to the apparatus influenced the insect inserting its antennae into the static electric field; the antennae that touched the G-MN were positively polarised and were temporarily the pole opposite the N-PIP (Figure 4B). Lowering the voltage applied to the N-PIP reduced the potential difference between the poles (charged plate and antenna tip), which reduced the range of the repulsive force of the N-PIP, and the antennae were inserted farther. When the tip of an antenna reaches a point with sufficient field intensity, the antenna is subject to an attractive force from the N-PIP that causes the insect to withdraw its antennae reflexively (Figure 4B). These findings support our hypothesis that the cockroach perceives an attractive force applied to the antennae introduced into the static electric field via electron deprivation from the antennae.

The structure of the present apparatus was simple and, therefore, easy to fabricate with common materials at low cost. Importantly, avoidance behaviour is common to wide-ranging insect species; as much as we tested, all of the insects possessing antennae showed the avoidance against the S-EF by inserting them into the field, which could be constructed by arraying a charged insulated conductor and a grounded metal net. Especially the grounding of the conductor net, which was the device used to flow the insect’s electricity to the ground, was an essential configuration for effectively repelling the insects that touch the net with the antennae. Thus, the present study provided an experimental basis for developing an electrostatic tool for excluding insect pests from protected spaces such as greenhouses, warehouses, animal husbandry facilities, hospitals and domestic houses.

## 4. Conclusions

Avoidance of a static electric field is a habitual insect behaviour that is harnessed to develop electrostatic apparatuses for pest control. This study examined an electrostatic event that caused the test insects (adult Turkestan cockroaches) to avoid entering the static electric field. Removing free electrons from the tip of the antennae inserted into the field triggered the subsequent positive electrification of the antenna tip, which was subject to the attractive force of the opposite pole. The attractive force pulled the antennae inside, while the insect pulled them back reflexively and moved backward. This action was recognised as insect avoidance behaviour. Thus, this study provides an experimental basis for analysing insect avoidance of a static electric field.

## Supplementary Materials

The following are available online at https://www.mdpi.com/article/10.3390/insects12070621/s1, Figure S1: Adult female (left) and male (right) Turkestan cockroaches used in this study, Video S1: (A) Avoidance behaviour by adult female (lower) and male (upper) Turkestan cockroaches released in the square surrounded by four S-EFPs charged with −6 kV. (B) Non-avoidance of the electric field of the S-EFP by a cockroach when the ground line of a galvanometer was removed to impede the flow of the insect-mediated transient electric current to the ground.
