# Supplementary figures and images for: Turkestan Cockroaches Avoid Entering a Static Electric Field upon Perceiving an Attractive Force Applied to Antennae Inserted into the Field
Source: Insects. 2021 Jul 8;12(7):621. doi: 10.3390/insects12070621 (PMC8303521; doi:10.3390/insects12070621)

**Figure S1**

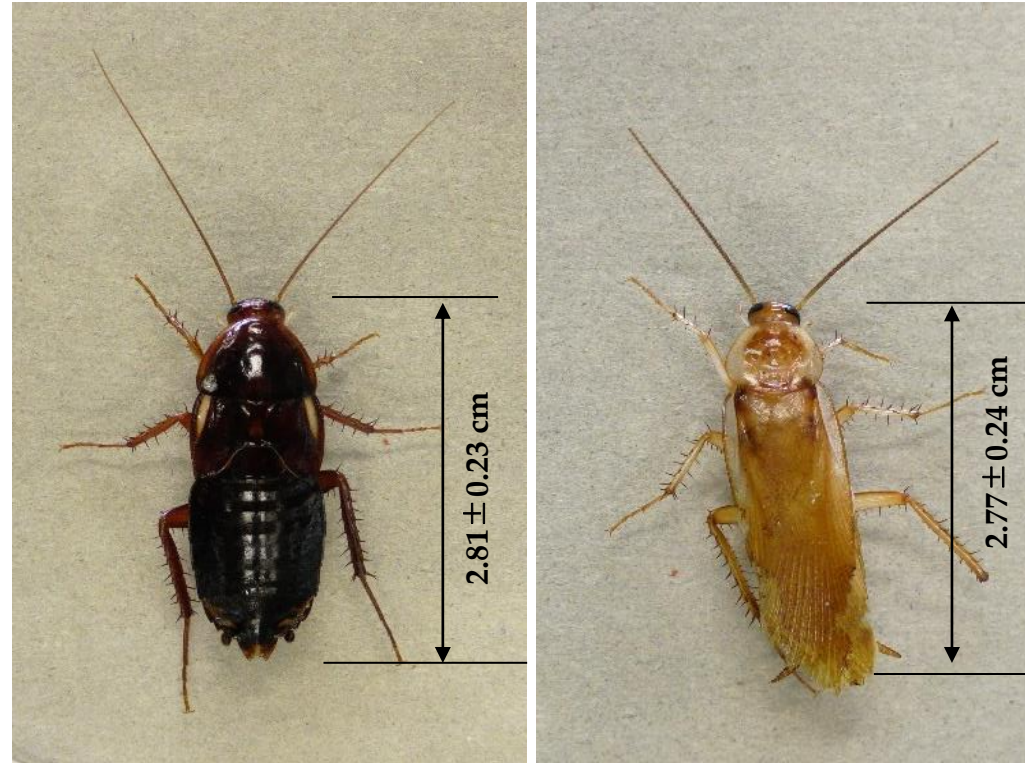

**Figure S1.** Adult female (left) and male (right) Turkestan cockroaches used in this study.

Supplement: Supplementary file 1 [file insects-12-00621-s001.zip › Figure S1.pdf]
